# Malformation of Tear Ducts Underlies the Epiphora and Precocious Eyelid Opening in *Prickle 1* Mutant Mice: Genetic Implications for Tear Duct Genesis

**DOI:** 10.1167/iovs.61.13.6

**Published:** 2020-11-03

**Authors:** Jiali Ru, Dianlei Guo, Jiaying Fan, Jiao Zhang, Rong Ju, Hong Ouyang, Lai Wei, Yizhi Liu, Chunqiao Liu

**Affiliations:** 1 State Key Laboratory of Ophthalmology, Zhongshan Ophthalmic Center, Sun Yat-sen University, Guangzhou City, China; 2 Guangzhou Woman & Children's Medical Center, Guangzhou City, China

**Keywords:** tear duct, Prickle 1, Wnt/PCP components, tear drainage obstruction, epiphora

## Abstract

**Purpose:**

Obstruction of the tear drainage causes a range of ocular surface disorders. Hitherto, the genetics of tear duct development and obstruction has been scarcely explored, and related animal models are lacking. This study aims to study the potential role of the Wnt/PCP pathway mediated by *Prickle 1* in tear duct development and diseases.

**Methods:**

A severe hypomorphic *Prickle 1* mutant was generated. Histology and immunohistochemistry were performed to compare wild type, *Prickle 1* hypomorphic, and null mutant tear ducts. In situ hybridization was conducted to identify the signaling components in the developing tear ducts. Three-dimensional (3D) reconstruction was used to detect the human embryonic tear duct.

**Results:**

Here, we report that a severe *Prickle 1* hypomorph mouse line exhibited epiphora. This phenotype was due to the blockage of the tear drainage by incompletely formed nasolacrimal duct (NLD) and lacrimal canaliculi (LC), which also causes precocious eyelid opening. We observed a dose-dependent requirement of *Prickle 1* for tear duct outgrowth. An investigation of the expression of Wnt/PCP core genes demonstrated a subset of PCP signaling components expressed in the developing tear duct. Furthermore, *Prickle 1* is not required for the expression of *Fgfr2/Fgf10* and *p63* genes, which are associated with the NLD and LC hypoplasia in humans. Last, we showed that *Prickle 1* expression in the developing tear drainage system is conserved between mice and humans.

**Conclusions:**

The study suggests that malformed tear ducts caused by disruption of *Prickle 1* underlies the epiphora and precocious eyelid opening.

A major function of the nasolacrimal apparatus in terrestrial animals is to keep the ocular surface from drying. It comprises two systems: the orbital glands and the excretory/drainage conduits, each with multiple components. Compromised glandular secretions or tear drainage leads to a range of ocular surface disorders, including the most common, dry eye disease, whose underlying genetics is barely understood.

The lacrimal canaliculi (LC) and nasolacrimal duct (NLD) are tear drainage conduits opening at the orbital and nasal epithelia, and are present in many tetrapods.^1^ The ocular fluid passes across the cornea and conjunctiva and drains into the nasal cavity through the LC and NLD.^2^^,^^3^ The NLD also possesses an absorptive function for tear fluid substances, which may provide a feedback signal in tear production, and thus, being associated with dry eye disease.^4^^,^^5^ Phylogenic variations of drainage ducts exist extensively among species.^6^^–^^9^ The human and rabbit NLD share similar tissue properties,^1^^,^^5^^,^^10^ but are different in origin, with the former originating from the ectodermal lacrimal groove and the latter from the subcutaneous region of the lower eyelid.^8^ The rabbit is considered a suitable model for human tear duct studies.^5^^,^^11^ The mouse NLD is suggested to develop similarly to that of humans based on a scanning electron microscopy study.^9^ However, little is known about the anatomy, genetics, and developmental biology of mouse NLD.

Clinically, congenital nasolacrimal duct obstruction (CNLDO) is estimated to be present in up to 20% of newborn infants,^12^^,^^13^ often causing epiphora with dacryocystitis and conjunctivitis. The genetic risk factors for CNLDO are poorly known. There are scattered reports on syndromic diseases, suggesting the existence of genetic elements underlying CNLDO.^14^^–^^20^ For instance, human mutations in fibroblast growth factor (FGF) signaling component FGF10, and its FGF receptors (FGFRs) 2 and 3 lead to Lacrimo-auriculo-dento-digital (LADD) syndrome, exhibiting hypoplastic NLD and puncta, often together with conjunctivitis. The secreted FGFs are important morphogens that play diverse roles during embryogenesis. The binding of FGFs to their FGFRs initiates signaling cascades participating in the morphogenesis of almost all tissues.^20^^,^^21^ In accordance with these findings, mutations in tumor protein p63 (transformation-related protein 63),^22^ a transcription factor upstream of *Fgfr2* gene,^23^ cause ankyloblepharon-ectodermal dysplasia-cleft lip/palate (AEC) syndrome, with lacrimal duct agenesis overlapping with LADD.^24^ Nonetheless, the genetic etiology of nonsyndromic CNLDO has not been uncovered, and CNLDO genetics is currently barely known.

Mammalian Wnts (wingless-type mouse mammary tumor virus [MMTV] integration site) constitute a large family of 19 secreted proteins, signaling through 10 Frizzled G-protein coupled receptors and other types of receptors and coreceptors with all possible combinations. The outcomes of Wnt signaling are manifested in a variety of biological processes, from the specification of cell fate to the generation of tissue/cell polarity, crucial for embryogenesis and organogenesis.^25^^,^^26^ The Wnt pathway is broadly divided into two branches: β-catenin-dependent (canonical) and -independent (non-canonical) Wnt signaling. Notably, non-canonical Wnt/PCP (planar cell polarity) directed epithelial polarity plays an indispensable role in tissue morphogenesis, including in gastrulation, tubule elongation, body fissure closure, inner ear hair cell orientation, and ciliogenesis, among others.^27^^–^^34^ The Wnt/PCP module consists of a set of six proteins, including Frizzled, Disheveled, Vangl, Prickle, Diego, and Flamingo, originally identified in Drosophila, which are crucial for coordinated hair and ommatidia planar orientations.^35^ Each protein has multiple orthologs in mammals with both overlapping and distinct functions.

We and others have generated null and hypomorphic mutant alleles of one of the mouse *Prickle* orthologs*, Prickle 1*.^31^^,^^36^^,^^37^ Depending on the genetic background, *Prickle* 1 null mutants exhibit either early lethality prior to gastrulation^36^ or pleiotropic defects, leading to death on the early postnatal days.^31^^,^^37^^,^^38^ Homozygous hypomorphs and compound null/hypomorphic mice survive normally, with a shorter stature, flat nose, and a short snout.^31^ We recently reported that *Prickle 1* mutant mice display delayed embryonic eyelid closure^39^ and precocious eyelid opening associated with ocular surface inflammation.^40^ The primary cause of the latter has yet to be identified. More recently, we observed watery eyes (epiphora) in *Prickle 1* compound null/hypomorphic mice, leading to our prediction that the mutant tear duct might be malformed, blocking the tear passage. In the current report, we present evidence that *Prickle 1* is required for normal tear duct development, malformation of which triggers epiphora, precocious eyelid opening, and ensuing ocular surface pathogenesis. Further investigations demonstrated that the Prickle gene family is also expressed in the human tear drainage system during development, suggesting that *Prickle 1* might be an important genetic contributor to CNLDO.

## Materials and Methods

### Mice and Genotyping

Animal husbandry and experimentation were conducted in strict adherence to the Standards in Animal Research: Reporting of In Vivo Experiments (ARRIVE) guidelines, the ARVO Statement for the Use of Animals in Ophthalmic and Vision Research, with approval from the Animal Care and Use Committee (ACUC) at Zhongshan Ophthalmic Center, Sun Yat-sen University. The severe *Prickle 1* hypomorph mutant line used in this study was generated by crossbreeding a *Prickle 1* gene-trap mutant allele (*Prickle 1^a/+^*) to a straight knockout allele (*Prickle 1^b/+^*).^31^^,^^40^^,^^41^ Mouse genotyping was conducted as described previously.^31^^,^^41^ Mouse strains were initially mixed genetic backgrounds of C57BL/6 and Sv129, backcrossing to C57BL/6 for multiple generations (> 7).

### Human Embryos

The human embryonic materials were provided by the Guangzhou Women and Children's Medical Center. The specimens were obtained from miscarriages and verified no congenital malformations. All human study protocols were reviewed and approved by the Institutional Review Board of the Guangzhou Women and Children's Medical Center.

### Histology

For fresh frozen sections of the mouse heads, postnatal mice (postnatal day = P1, P5, and P8) were euthanized by decapitation and adult mice were euthanized by cervical dislocation. The dissected heads were directly embedded in OCT (Cat. 4583; SAKURA, USA) and frozen in liquid nitrogen and store at -80°C in the freezer. Sections were cut at 15 µm for all immunostaining purposes except for 3D reconstruction, in which 30 µm sections were prepared.

For paraffin eyeball sections, the eyeballs, including the intact eyelids, were dissected and fixed in 4% paraformaldehyde for 24 hours at 4°C.^42^ The samples were washed three times in PBS, dehydrated through a series of alcohols and three times in xylene, then embedded in paraffin and sectioned using microtome (Leica RM 223; Wetzlar, Hesse-Darmstadt, Germany). Hematoxylin and eosin (H&E; Cat. G1002 and Cat. G1004; Servicebio, China) staining followed the manufacturer's instructions.

For prefixed frozen sections, mouse heads were bisected and paraformaldehyde (PFA)-fixed, as stated before. After PBS washes, tissues were submerged in 30% sucrose overnight for cryoprotection, embedded in OCT, and stored at -80°C before sectioning.

### Immunohistochemistry

For frozen sections and fresh frozen sections, immunostaining followed a standard protocol. Briefly, tissue sections were blocked with 10% donkey serum with 0.1% Triton X-100 in PBS (PBST) for 30 minutes at room temperature (RT), then incubated with primary antibodies at 4°C overnight. After being washed with PBST, the sections were incubated with the fluorescent dye-conjugated second antibodies for 1 hour at RT, washed with PBST, and mounted with Fluoromount-G (Southern Biotech, Birmingham, AL, USA). Antibodies used in this study are anti-E-cad (ab11512; Abcam) and anti-p63 (ab124762; Abcam).

### In Situ Hybridization

Digoxigenin (DIG; 11277073910; Roche) DIG-labeled sense and antisense RNA riboprobes were prepared by in vitro transcription with T7 and T3 RNA polymerase (T3: M0378S and T7: M0251S; BioLabs) using T7 (5′-TAATACGACTCACTATAGGG-3′) and T3 (5′-AATTAACCCTCACTAAAGGG-3′) promoter-tagged PCR fragments obtained from each gene. Primers used for PCR are listed in Supplementary Table S1. In situ hybridization (ISH) was performed using a protocol that was described by Jensen and Wallace.^43^

### Tear Fluid Collection, Protein Gel, and Western Blot Analysis

Tears were induced by intraperitoneal injection of pilocarpine (300 µg/kg body weight) and collected from the eyelid margin into Eppendorf tubes using a 0.5 µl micropipette 5 minutes after injection. The same volume of tears from wild type and the mutant mice were used for running a 10% SDS-PAGE. Protein profiles were visualized by Coomassie brilliant blue staining for 2 hours at RT followed by destaining with 40% methanol and 10% glacial acetic acid. For Western blot analysis, protein samples were blotted onto polyvinylidene difluoride (PVDF) membranes by a wet transblot system (Mini-Protein Tetra; Biorad) using standard protocol recommended by the manufacturer. After blocking with 5% fat-free milk, membranes were incubated with primary antibody against mouse lactoferrin (07-685; Millipore) and then peroxidase-conjugated secondary antibody IgG (M21002S; Abmart, China). Chemiluminescent images were taken by FluorChem R (Proteinsimple).

### Imaging

Fluorescence microscopy images were obtained using Zeiss confocal microscope (Zeiss LSM880; Zeiss, Oberkochen, Germany) and Imager.Z2 equipped with ApoTome (Zeiss). H&E and ISH images were acquired by Imager.Z2.

### 3D Reconstruction of Tear Duct

3D

Images taken from microscopes were aligned manually by Photoshop software according to anatomic features of each section. NLD structure was traced on each section and imported to NIH ImageJ software for 3D processing (with 3D viewer plugins) with properly set image and pixel depth and adjusted image coordinates.

### Quantification and Statistics

For measuring the missing length of NLD, coronal sections from P1 wild type, severe *Prickle 1* hypomorph, and null mutant heads were subjected to H&E staining. The first section displaying NLD in both wild type and the mutant mice was designated as “zero NLD length,” respectively. Missing NLD in the mutants was calculated as vertical distances between the section at “zero NLD length” and the section exhibiting the most similar anatomic structures to that of wild type at “zero NLD length” (missing NLD length = section thickness × no. of sections). Six animals were used for each genotype. Student *t*-test was used to detect the power of significance.

## Results

### Tear Duct Dysplasia in Severe *Prickle 1* Hypomorphic Mutants Led to Epiphora

**Figure 1.**
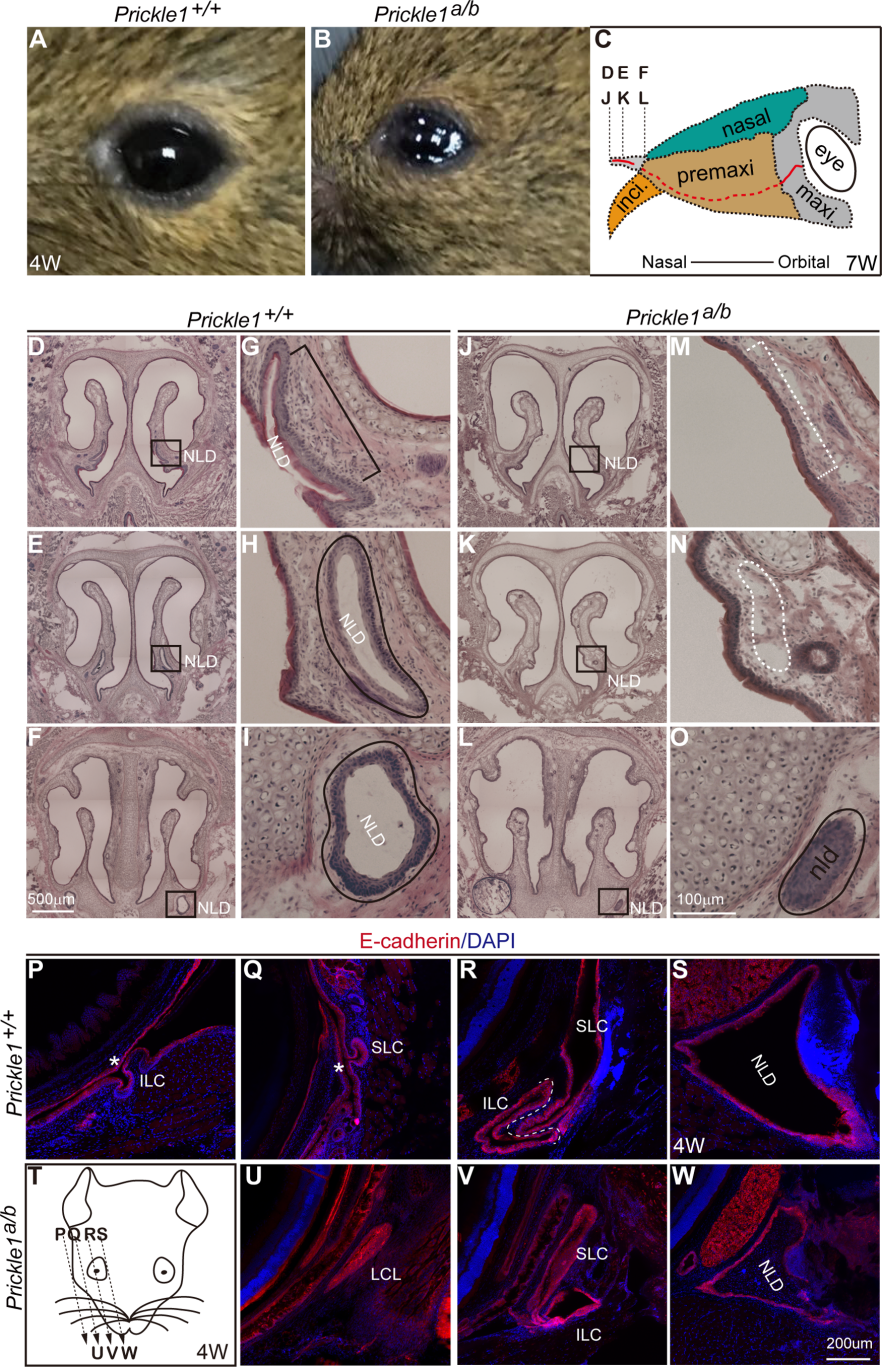
The tear duct histology and epiphora in the severe *Prickle 1* hypomorph mutants. Abbreviations apply to all figures unless otherwise noted. Fresh frozen sections were subjected to H&E and immunohistochemistry. (**A**) Normal cornea surface. (**B**) Mutant cornea surface exhibiting epiphora. (**C**) Section positions in (**D–F**) and (**J–-L**). **D** to **F** Wild type nasal sections. Boxed areas are the nasolacrimal duct (NLD). (**G–I**) Magnified from **D** to **F**. Bracket and lines indicate the NLD. **J–L** Mutant nasal sections positionally correspond to that of the wild type in **D** to **F**). Boxed areas are magnified in **M** to **O**. **M–O** Dashed brackets and lines indicate the presumptive NLD. (**P–W**) The E-cadherin staining revealed the NLD and the canaliculi. (**P–S**) Representative sections of wild type tear duct from different positions. Sectioning direction is from temporal to nasal as illustrated in (**T**). SLC, superior lacrimal canaliculus; ILC, inferior lacrimal canaliculus; Asterisks, canaliculi openings at eyelids. (**U–W**) Mutant tear duct sections from temporal to nasal.

A severe hypomorphic *Prickle 1* mutant with compound heterozygous null and hypomorphic alleles was generated (see Materials and Methods, Supplementary Fig. S1A).^39^^,^^40^ We estimated that the levels of expression of *Prickle 1* were lower than 25% of the wild-type levels on Western blots of limb and brain tissues (Supplementary Figs. S1B, S1C). This severe hypomorph exhibited epiphora in adulthood (Figs. 1A, 1B). We suspected that the tear drainage was obstructed, prompting us to examine the tear duct histology. Transverse fresh-frozen sections from the nasal proximity of the mouse head (Fig. 1C) were subjected to H&E staining (Figs. 1D–O). Sections from wild type and mutant mice with the most similar anatomic structures were compared (Figs. 1D–F, 1G–I compared with Figs. 1J–L, 1M–O, respectively). The absence of the distal NLD was observed in all the examined mutant animals (Figs. 1J–O; *n* = 3). We next examined LC, which normally branches out from the orbital extreme of the primordial tear duct^10^ and opens at the palpebral conjunctiva in humans. Oblique sections parallel to the facial plane were prepared (Figs. 1P–W; *n* = 3). Using E-cadherin staining, the openings of both the upper and lower canaliculi to the front canthus were detected in wild-type mice (Figs. 1P, 1Q), but not in the mutants (Fig. 1U), even though all other corresponding structures could be observed in both wild type and mutant mice (Figs. 1R, 1S compared to Figs. 1V and 1W, respectively). Thus, the missing ends of the LC blocking tear drainage explain the epiphora phenotype.

### Tear Duct Dysplasia Coincided With Pooling of Tear Fluid Under the Eyelid in *Prickle 1* Hypomorphic Mutants

**Figure 2.**
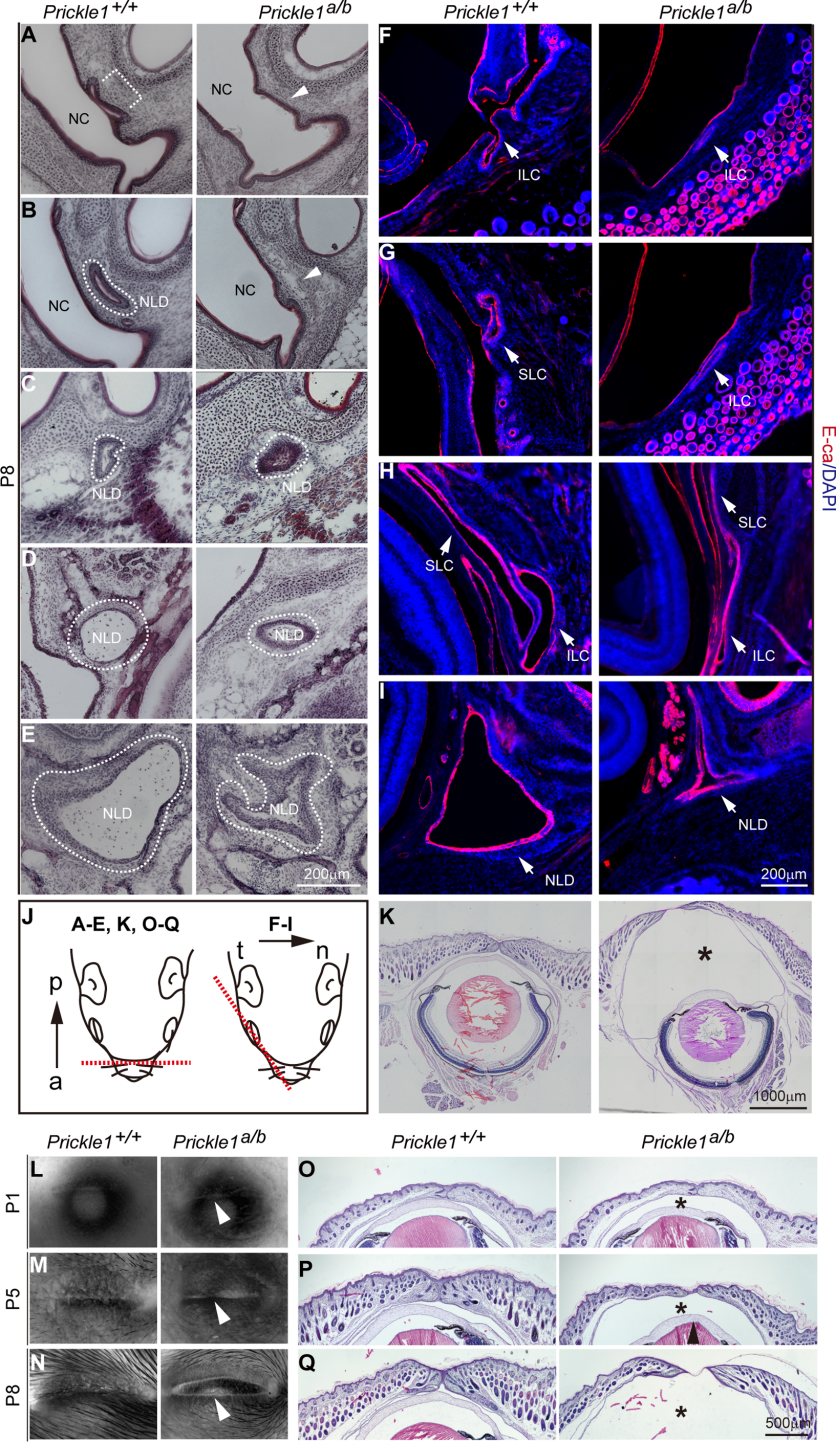
Tear duct obstruction, tear fluid retention and ruptured eyelid in the *Prickle 1* hypomorphic mutants. (**A–E**) H&E-stained fresh frozen sections. NC, nasal cavity; *Arrows* and *lines* indicate the NLD positions. (**F–I**) The E-cadherin staining revealed the NLD and LC. Note openings of the lacrimal canaliculi in the lower **F** and the upper **G** eyelid of wild type P8 mice were missing in the mutants. The furthest extended mutant SLC on the section is shown in right panel to **H**. *Arrows* point to different parts of the tear duct. (**J**) Section direction for **A** to **E**) (*left*) and **F** to **I** (*right*). a, anterior; p, posterior; t, temporal; n, nasal. (**K**) Whole-eyeball coronal sections at P8. The asterisk indicates the mutant ocular fluid accumulation under the eyelid. (**L–N**) Eyeballs viewed from the top. *Arrows* point to the mutant eyelid. (**O–Q**) H&E stained coronal sections of the eyelid.

The absence of nasal NLD and orbital LC suggested a developmental abnormality of the drainage system. Thus, we examined the early postnatal ages of mice to inspect the consistency of this phenotype. An examination of the NLD using H&E histology demonstrated a similar absence of the nasal end of the mutant NLD at postnatal day 8 (P8; Figs. 2A–C; *n* = 5), while preserving the medial part (Figs. 2D, 2E). Immunostaining for E-cadherin revealed missing orbital openings in the canaliculi (Figs. 2F, 2G right panels compared with the left), whereas the remaining LC connecting with NLD was generally preserved (Figs. 2H, 2I right panels compared with the left; *n* = 5). These data are consistent with the findings observed in adult mutant mice (see Fig. 1). Surprisingly, we observed a remarkable accumulation of ocular fluid under the eyelid, apparently pressuring the lid nearly to open (Fig. 2K; *n* = 6). This observation explains the mutant precocious eyelid opening we reported previously,^40^ and led us to further investigate mutant ocular fluid production in a time series.

No difference was detected between the mutant and wild-type eyelids from the top views of the P1 eyeballs (Fig. 2L; *n* = 4). The mutant lid appeared to be protuberant and smoother at P5 compared with that of the wild type (Fig. 2M, right panel; *n* = 5), and the stretched eyelid was conspicuous at P8, with the lid junction nearly broken (Fig. 2N, right panel; *n* = 6). On H&E-stained sections, increased ocular fluid in the mutants was reflected by the larger space between the cornea and the eyelid, which is obvious, even at P1 (Fig. 2O). The space progressively expanded until the eyelid ruptured (Figs. 2P, 2Q). These data suggest that the obstruction of tear drainage is caused by the malformed tear duct, which, in turn, results in mutant ocular fluid retention and, ultimately, in eyelid rupture.

### The Accumulated Ocular Fluid in the Mutants was Tears and the Mutant Ocular Glands were Largely Normal

**Figure 3.**
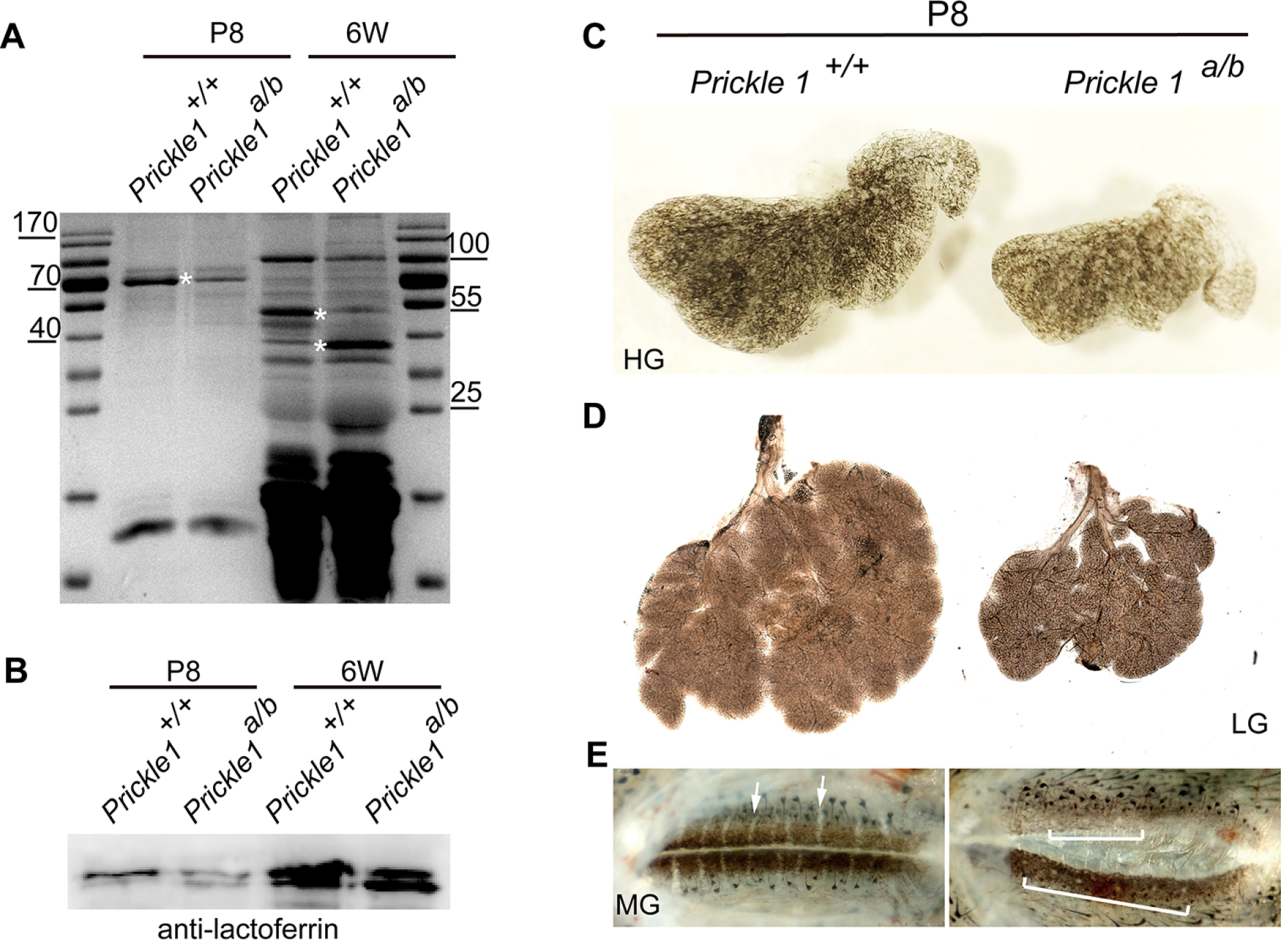
Accumulated ocular fluid in the *Prickle 1* hypomorphic mutants is primarily of tears. (**A**) SDS-page profiling the mutant and wild type tears at P8 and 6-week old (6W) animals. Asterisks indicate differential intensities of similar molecular shifts in the mutant and wild type tears. (**B**) Immunoblotting of the mutant and wild type tears using anti-transferrin antibody. (**C**) Wild type and the mutant P8 Harderin glands (HGs). (**D**) Lacrimal glands (LGs). (**E**) Meibomian glands (MGs) indicated by *arrows* and *brackets* respective for wild type and the mutant mice.

We hypothesized that if the accumulated mutant ocular fluid is due to the obstructed tear ducts, then it must be tears. To confirm this hypothesis, we compared mutant ocular fluid with wild-type tears at P8 before the eyelid opening. Because tears normally drain down to the naris, preventing the collection of a sufficient amount for analysis, pilocarpine was used to stimulate tear production in P8 wild-type mice. Tears were then collected from the eyelid margin. On the other hand, mutant ocular fluid could be readily collected with a syringe from the enclosed space under the eyelid. The same volumes of tears were run on an SDS denaturing gel. As predicted, the protein profiles were comparable between the mutant and wild-type mice at P8 (Fig. 3A). Next, we stimulated tear production in both 6-week-old wild type and mutant mice and performed the same analysis as we did for P8. Similar results were observed; no qualitative differences were detected on the SDS-PAGE gel (see Fig. 3A). Consistent with these observations, lactoferrin/transferrin, a major component of tears, was also expressed at a similar level in wild type tears and the mutant ocular fluid (see Fig. 3B). Therefore, we concluded that the mutant ocular fluid is primarily tears, and that the mutant lacrimal gland function is largely normal.

The accumulated tear fluid in the mutants might also result from excessive production of the ocular glands in combination with a dysplastic tear duct. Notwithstanding, the gross examination of the ocular glands at P8 showed normal general morphology of the mutant Harderin and lacrimal glands, except for smaller sizes, presenting the same number of lobes as that of wild-type mice (Figs. 3C, 3D, Supplementary Fig. S2; *n* = 3). Additionally, the Meibomian gland was aberrant in the mutants (Fig. 3E), which might be due to compression by the accumulated tears.

To examine whether *Prickle 1* plays a role in lacrimal gland branching, as several other PCP components (such as Vangl2), we first performed immunohistochemistry (IHC) using E-cadherin antibody to locate the epithelia of the lacrimal gland and canaliculi, and GFP antibody to identify *Prickle 1* expression on the same sections at E14.5 (Supplementary Figs. S2A–G; *n* = 3). Although a clear co-labeling fluorescent signal was present in the canaliculi (Supplementary Figs. S2E–G), no GFP expression was detected in the lacrimal gland (Supplementary Figs. S2B–D). Consistent with this observation, no branching defects were exhibited in the *Prickle 1* mutants at E16.5 (*n* = 3). These results suggest that, unlike some other PCP components, Prickle does not seem to function in the lacrimal gland.

### Dose-Dependent Requirement of *Prickle 1* for the Tear Duct Formation

**Figure 4.**
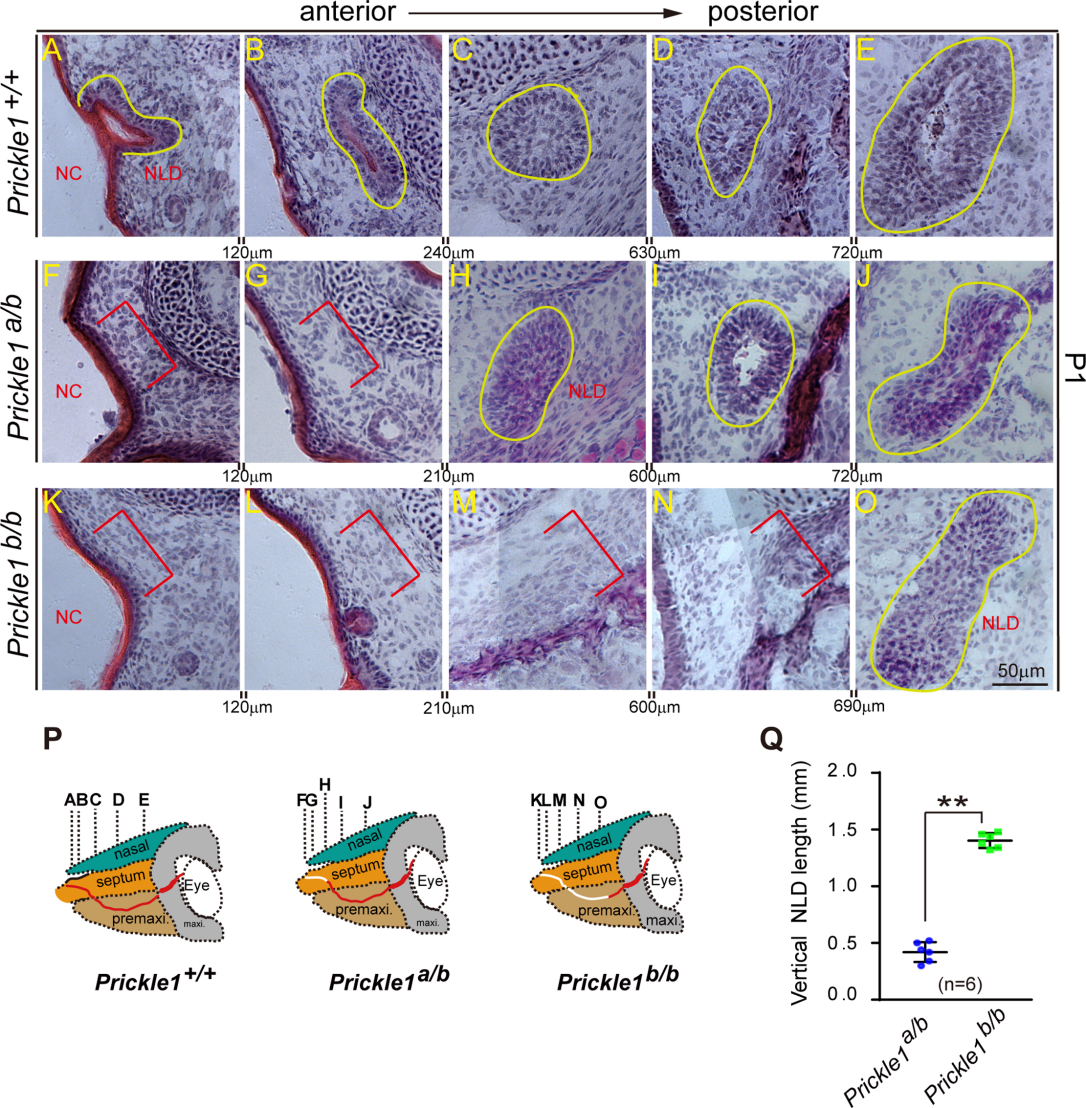
*Prickle 1* dose-dependent loss of the nasolacrimal duct. (**A–O**), H&E-stained fresh-frozen sections. *Yellow lines* indicate the existing NLD, and *red brackets* indicate missing of the NLD. Interval distances are shown between each panel. **A** to **E**) Wild type NLD at P1. **F** to **J** The *Prickle 1* hypomorphic mutant duct with null (*Prickle 1^b^*) and hypomorph (*Prickle 1^a^*) alleles. **K** to **O** The *Prickle 1^b/b^* null mutant duct. (**P**) Schematic illustration of dose-dependent loss of the NLD (*white lines*) on *Prickle 1* hypomorphic and null mutant alleles. (**Q**) Quantification of the missing NLD of the *Prickle 1^a/b^* and *Prickle 1^b/b^* mutants. The missing NLD was quantified as vertical distances (number of sections × section thickness) from the presumptive nasal ends to sections that first appear NLD. Student *t*-test was performed to detect significance.

In general, *Prickle 1* mutants with null alleles have more severe phenotypes than those with hypomorphic or null/hypomorph compound alleles. This is partially reflected by the fact that hypomorph/null *Prickle 1* compound mutants (*Prickle^a/b^*) survive normally, whereas null mutant (*Prickle^b/b^*) mice die within ∼ 24 hours after birth.^31^ We thus reasoned that *Prickle 1* null mutants might also have a more severe tear duct phenotype. Therefore, we compared the tear duct of different genotypes at P1. On H&E-stained sections, wild-type NLD completely reached and opened at the nasal cavity at P1 (Fig. 4A; *n* = 6) with a regional irregularity in tube shape (Figs. 4B–E). In contrast, the *Prickle 1^a/b^* and the *Prickle 1^b/b^* NLD missed 300 µm and 1.5 mm in vertical section thickness respective to their nasal cavities (Figs. 4F–O; *n* = 6). Quantification of the vertical NLD length demonstrated a significant dose-dependent NLD missing on the disruption of *Prickle 1* gene (Figs. 4P, 4Q).

### Expression of FGF and Wnt/PCP Signaling Components in the Developing Mouse Tear Duct

**Figure 5.**
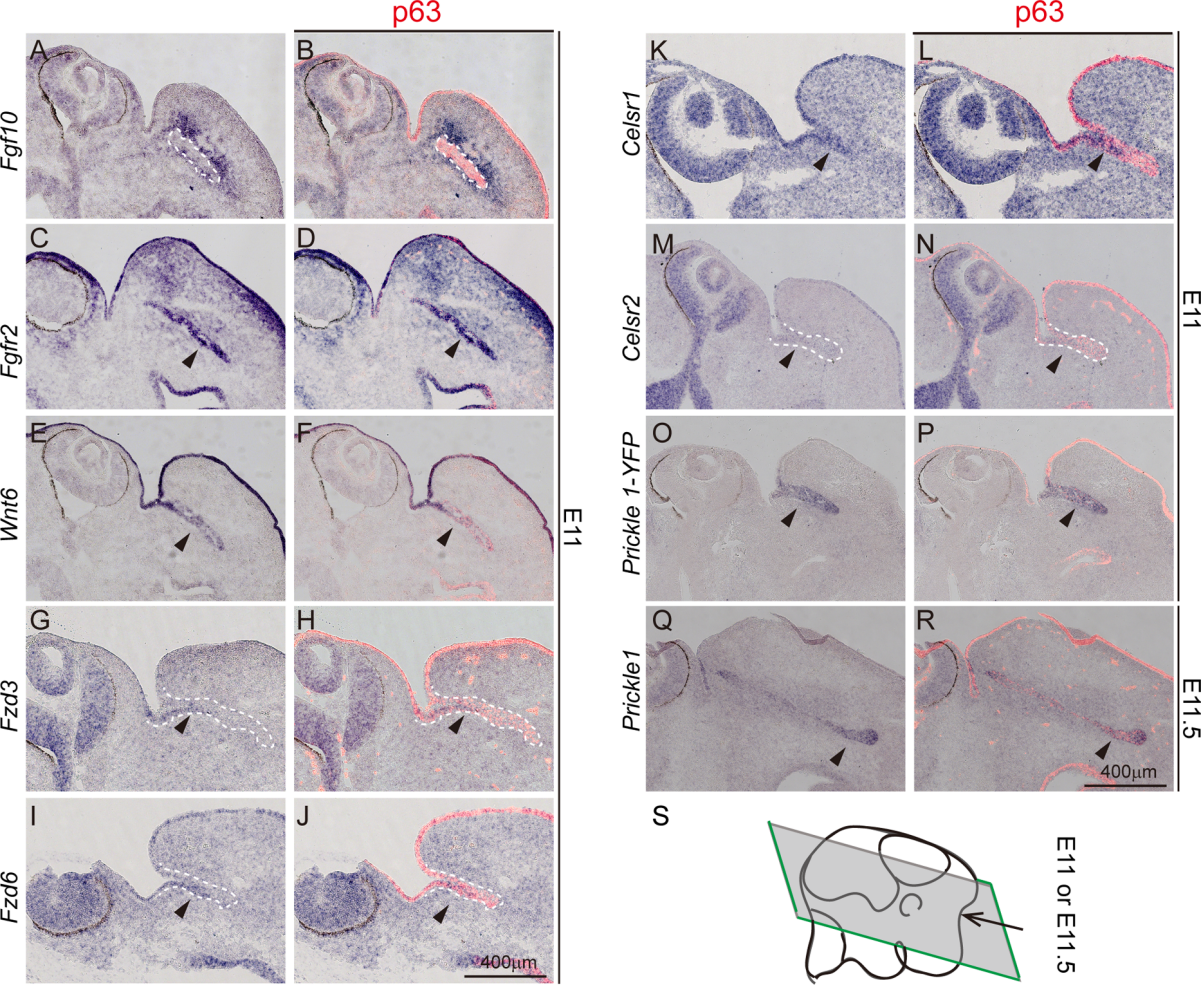
Expression of the *Wnt/PCP* and *Fgf* signaling components in the developing tear duct. All panels are in situ hybridization (*purple*) followed by p63 immunostaining (*red*). *Dashed lines* demarcate the tear duct, *arrows* indicate gene expression. (**A, B**), *Fgf10*. (**C, D**), *Fgfr2.* (**E, F**), *Wnt6*. (**G, H**), *Fzd3*. (**I, J**), *Fzd6*. (**K, L**), *Celsr1*. (**M, N**), *Celsr2*. (**O–R**), *Prickle 1*. (**S**) Schematic illustration of a horizontal sectioning plane through the developing tear duct at E11.

Because FGF and Wnt/PCP pathways are known to be extensively involved in duct morphogenesis, we investigated their expression in the early phase of tear duct development. We focused on embryonic day E11, when the growing tear duct could be identified from the eyelid and conjunctiva expressing epithelial marker p63 (Figs. 5A–P). *Fgf10* was expressed surrounding the tear duct (Figs. 5A, 5B) complementary to *Fgfr2*, which was expressed in the tear duct itself (Figs. 5C, 5D). In contrast, *Fgfr3* was not expressed in the tear duct, despite its strong expression in the lateral ventricles (Supplementary Figs. S3A–D). An investigation of the expression of the WNT/PCP components found that among the 19 Wnts, *Wnt6* was highly expressed in the tear duct and skin epithelium (Figs. 5E, 5F; Supplementary Fig. S3M), whereas other *Wnts* were only weakly expressed or did not have a distinct expression pattern in the tear duct (Table; Supplementary Fig. S3). Of the 10 Frizzled (Fz) receptors, *Fz3* and *Fz6* were weakly expressed in the initial parts of the tear duct (Figs. 5G–J, Table; Supplementary Fig. S4). Two of the three PCP atypical cadherins, *Celsr 1* and *Celsr 2*, were expressed in the initial and full tear ducts, respectively (Figs. 5K–N, Table; Supplementary Fig. S5A). Interestingly, none of the *Dvl*, *Vangl* families, or *Inversin* was distinctly expressed in the developing tear duct (Supplementary Fig. S5). As expected, *Prickle 1*, but not other Prickle family members, was restricted to the tear duct indicated by an antisense probe against *eYFP* reporter gene (Figs. 5O, 5P; Supplementary Fig. S6). The reporter expression was verified using a *Prickle 1* antisense probe (Figs. 5Q, 5R [E11.5]). Taken together, the expression of FGF signaling components is consistent with the NLD and LC phenotypes observed in human patients bearing *Fgf10/Fgfr2* mutations, and the six PCP core genes do not seem to work together all the time.

**Table.** A Summary of PCP Components Expressing in Early Tear Duct Using In Situ Hybridization

Wnt/PCP Family	Tear Duct	Surrounding Tear Duct	Uniformly or No Expression
*Wnt*	*Wnt1*, *4*, *6*	*Wnt11*, *5a*	*Wnt2*, *2b*, *3*, *3a*, *5b*, *7a*, *7b*, *8a*, *8b*, *9a*, *9b*, *10a*, *10b*, *16*
*Prickle*	*Prickle 1*	*–*	*Prickle 2*, *3*, *4*
*Frizzled*	*Fzd 3*, *6*	*Fzd 10*	*Fzd 1*, *2*, *4*, *5*, *7*, *8*, *9*
*Dvl*	*–*	*–*	*Dvl 1*, *2*, *3*
*Celsr*	*Celsr1*, *2*	*–*	*Celsr3*
*Vangl*	*–*	*–*	*Vangl1*, *2*
*Inversin*	*–*	*–*	*Inversin*

### Disruption of *Prickle 1* did not Alter *p63*, *Fgf10*, *Fgfr2*, or *Wnt6* Expression

**Figure 6.**
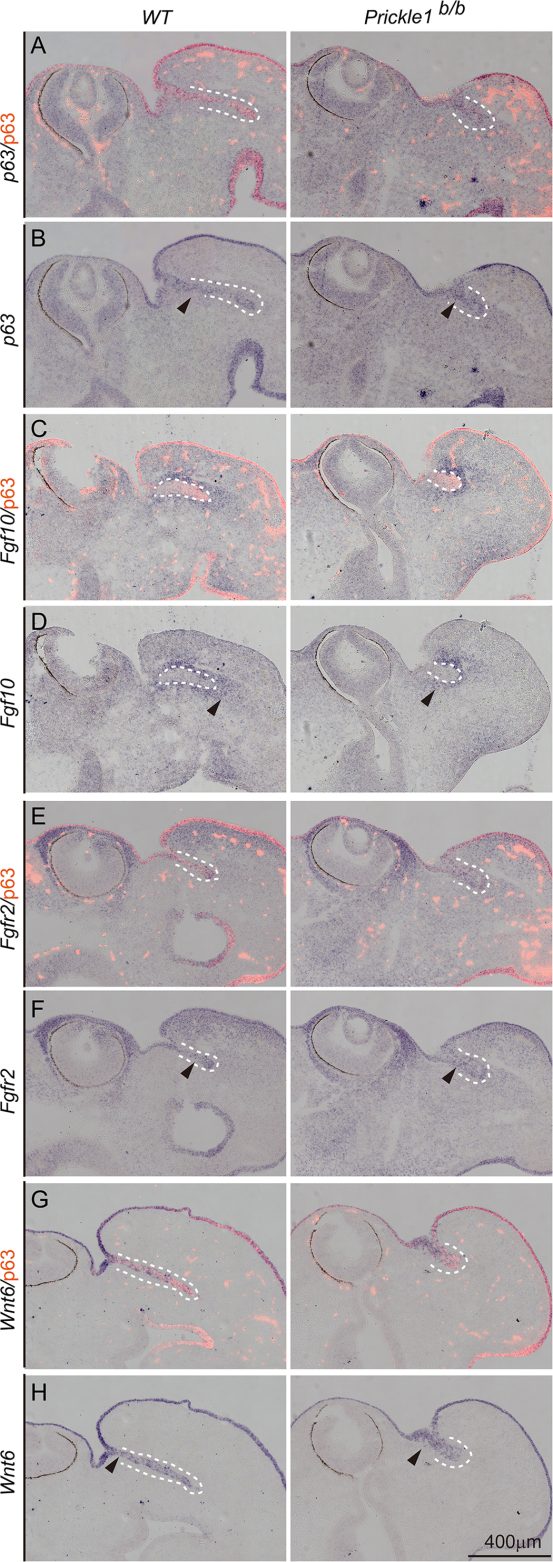
Expression of *Fgfr2*, *p63*, *Fgf10*, and *Wnt6. Dashed lines* demarcate the tear duct, *arrows* indicate gene expression. All panels are in situ hybridization (*purple*) followed by p63 immunostaining (*red*). The right column of panels is from wild type embryos, whereas the left column of panels is from the *Prickle 1* null mutants. (**A, B**), p63. (**C, D**), *Fgf10*/p63. (**E, F**), *Fgfr2*. (**G, H**), *Wnt6*.

The distinct expression of *Wnt6*, *p63*, *Fgf10*, and *Fgfr2* in the tear duct prompted us to ask whether their expression would be altered in *Prickle 1* mutant mice. To address this, we used wild type and the *Prickle 1* null mutant embryos for examination. The outgrowth of the tear duct was apparently stunted at the examined age of E11 (Figs. 6A–H). In situ hybridization showed no remarkable difference in the expression of *p63* (Figs. 6A, 6B), *Fgf10* (Figs. 6C, 6D), and *Fgfr2* (Figs. 6E, 6F) between wild type and *Prickle 1* mutant mice. *Wnt6* expression also remained similar in the wild type and mutant tear ducts (Figs. 6G, 6H). Thus, these results suggest that, genetically, *Prickle 1* is either downstream or parallel to FGF signaling during tear duct growth.

### Expression of Prickle Gene Family in the Human Tear Duct Development

**Figure 7.**
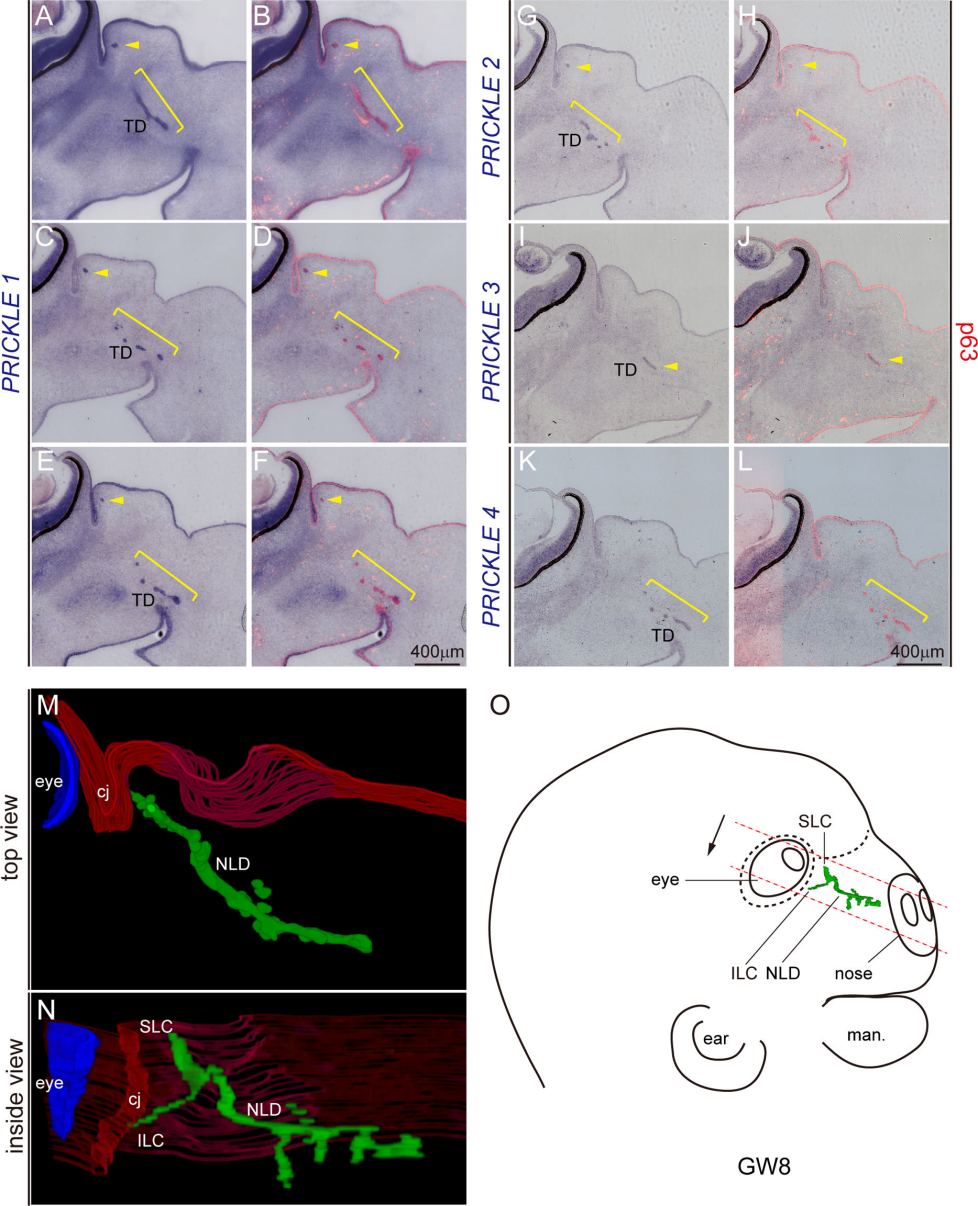
Expression of Prickle family in the developing human tear duct. (**A–L**) All panels are in situ hybridization (*purple*) followed by p63 immunostaining (*red*) at gestation week 8 (GW8). *Brackets* and *arrows* indicate areas of the developing tear duct (TD) and in situ hybridization signal. **A**, **C**, and **E** are three consecutive sections with 30 µm intervals. **B**, **D**, and **F** are same sections respective to **A**, **C**, and **E**. **A** to **F** *PRICKLE1/*p63. **G** and **H**) *PRICKLE2/*p63. **I** and **J**) *PRICKLE3/*p63. **K** and **L** *PRICKLE4/*p63. **M** and **N**) The 3D reconstruction of the human embryonic tear duct with p63 staining. The tear duct is drawn as *green*, whereas the head surface epithelium is drawn as *red*. **M** Top view of the developing tear duct. **N** Inside view of the developing tear duct. cj, conjunctiva; NLD, nasolacrimal duct; SLC, superior lacrimal canaliculus; ILC, inferior lacrimal canaliculus. (**O**) Schematic illustration of developing TD in the context of the GW8 embryonic head. *Red dashed lines* and *black arrows* indicate sectioning region and direction, respectively. man, mandibulary.

We predicted that the function of *Prickle 1* in tear duct development might be conserved in mammals. It is especially of interest to see this in humans to explore *Prickle 1* mutant mice as potential disease models. Therefore, we investigated the expression of the Prickle gene family in the development of the human tear duct. We collected gestational 8-week-old (GW8) embryos, when the lacrimal groove was formed via the fusion of the lateral nasal and maxillary.^10^ Using ISH, followed by immunostaining of p63, *Prickle 1* was highly expressed in the developing human tear duct (Figs. 7A–F). Interestingly, unlike the mouse, human Prickle 2, 3, and 4, although weakly, were also expressed in the developing tear duct with distinct patterns (Figs. 7G–L). Furthermore, all *Prickle* genes exhibited unusual dotted expression patterns, prompting us to examine the path of the developing human tear duct. Using p63 as an epithelial marker, we reconstructed images from all embryonic sections comprising the fetal tear duct at GW8. On the 3D developing duct, multiple tubular branches were found (Figs. 7M, 7N; Supplementary Movie S1). These branches generally extended in the vertical plane because the top view of the 3D image missed most of them (see Fig. 7M). This pattern might reflect an unsynchronized pinching off of the regional lacrimal groove epithelial cells, which migrated downward, collectively and independently. A diagram of NLD fitted with a GW8 embryonic head is shown in Figure 7O.

## Discussion

Tear drainage obstruction leads to a range of ocular disorders, often with epiphora, keratoconjunctivitis, and dacryocystitis. Despite its importance, little attention has been paid to this system, probably because (1) tear duct obstruction is not a life-threatening disease; therefore, insufficient genetic data have been collected in clinics; (2) it is entirely embedded in the complex bony and cavernous tissues, restricting its accessibility in research; and (3) no suitable animal models are available for human studies. In an attempt to fill these blanks, the current study demonstrates how tear drainage obstruction could lead to a chain of ocular surface disorders using a genetically engineered mouse model, which is potentially useful for related human diseases. We further extend our investigations to previously unexplored genetic determinants of the tear duct, providing a framework for the future understanding of developmental biology and diseases of the drainage system. Our work additionally provides a potential tear drainage-related human disease model, offering opportunities to explore novel biological functions of PCP.

In a previous study, we demonstrated that embryonic eyelid closure is delayed in *Prickle 1* null/hypomorph compound mutants, but the lids completely joined before birth.^39^ In a separate study, we showed that the mutant eyelid reopens prematurely, which is associated with ocular surface inflammation, likely caused by the necrotic eyelid tissue falling on the ocular surface.^40^ Precocious eyelid reopening is probably independent of early delayed eyelid closure, judging from the time period between the two events. Additionally, the ocular surface, including the eyelid, appears to develop normally in the mutants, as keratin markers are correctly expressed in the perinatal and early postnatal ocular surface before the eyelid reopens.^39^ Thus, the mechanism underlying the previously identified precocious eyelid reopening remains an enigma. The current study suggests that the malformed tear duct in the *Prickle 1* mutants is likely the culprit for the advanced eyelid reopening and the associated ocular surface pathogenesis.^39^^,^^40^ In normal mice, the lacrimal gland starts secreting tears already at P1, which drain through the tear conduits to the naris. In the *Prickle 1* mutant mice, however, although tears are normally produced, they fail to drain out. The continuous production of the tear fluid without drainage builds up pressure against the enclosed eyelid, eventually forcing it to open. The ruptured eyelid debris then falls back to the corneal and conjunctival surfaces, further eliciting keratoconjunctivitis.^40^ However, alternative interpretations still exist because the impact of Prickle-1-mediated PCP on corneal development is yet to be investigated in detail. Additionally, whether the cross-talk between the eyelids and ocular surface is impaired in the *Prickle 1* mutants, and how it may contribute to the mutant corneal pathology remains to be addressed.

Although coevolved, the lacrimal gland develops much later than the tear duct. *Prickle 1* seems to have a more specific role in tear duct morphogenesis than in the ocular glands. Indeed, the morphology of the ocular glands is largely normal in the *Prickle 1* mutants, and the tear components do not change much either. The primary role of *Prickle 1* in the tear duct could also be reflected from the dosage-dependent phenotypic manifestation of the mutant NLD. Anatomically, the NLD can be roughly divided into three segments along the head axis: orbital/proximal, bony/middle, and narial/distal.^7^^,^^44^ The *Prickle 1* heterozygous compound mutants miss only partial distal NLD, whereas null mutants miss more. The graded phenotypic severities suggest that the role of *Prickle 1* in the tear duct is rather specific and primary.

Because *Prickle 1* is a key component of the Wnt/PCP core protein complex, we investigated the expression of all known Wnt/PCP components*.* In addition to the confirmation of *Prickle 1* expression in the tear duct, we found that not all PCP core complex genes were expressed together with *Prickle 1*. This further suggests that tissue context-dependent combinations of PCP core members coordinate the morphogenesis of different tissues. Among the 19 *Wnts*, only *Wnt6* is highly expressed in the tear duct. However, whether it transduces signals through β-catenin-dependent or PCP pathways requires further investigation.

Three FGF signaling components, *Fgfr2*, *3*, *Fgf10*, and p63, an upstream transcription factor for *Fgfr2*, are known to be involved in human LADD syndrome^20^^,^^23^^,^^24^ with obstructed NLD and LC. Consistently, all of them are expressed in the mouse tear duct or its surrounding tissues, except for *Fgfr3*. It is appealing to know whether Wnt/PCP would genetically interact with FGF signaling. However, we did not observe remarkable changes in the expression of the examined FGF components in the *Prickle 1* mutants, implying that Prickle 1-mediated PCP functions are either parallel or downstream of FGF signaling. Notably, the *Fgf10/Fgfr2* pair also plays an essential role in lacrimal gland development,^45^ which is barely affected in *Prickle 1* mutant mice. Therefore, FGF and Wnt/PCP may operate on different aspects of tear duct development. Nonetheless, whether the Wnt/PCP pathway is downstream of FGF signaling is yet to be clarified.

Comparative studies revealed variations in both tear duct anatomy and origin among species.^1^^,^^5^^–^^9^^,^^11^^,^^46^ It is suggested that the rabbit NLD, rather than the rat NLD, shares similar tissue properties with that of humans, and is considered a suitable model for the study of NLD substance absorption.^5^^,^^11^ However, unlike humans, the rabbit NLD originates from the subcutaneous region of the lower eyelid,^8^ rather than the junction epithelia, the nasolacrimal groove formed by the maxillary and nasal processes.^10^ In contrast, both the rat and mouse NLD initiate from the nasolacrimal groove as the human does.^9^^,^^10^^,^^47^^,^^48^ Thus, it appears that the similarity in tear duct development among species does not necessarily reflect the similarity of the tear duct physiology. Genetically, *Prickle 1* expression in both human and mouse tear ducts implies its evolutionarily assigned molecular function in this organ. However, unlike the mouse, other Prickle members were also weakly expressed in the developing human tear duct. Morphologically, multiple branches of the primordial tear duct observed in humans are not observed in mice. Thus, despite the conserved *Prickle 1* expression and the similar NLD origin between mice and humans, further studies are required to examine whether the mouse is a suitable animal model for studying human NLD-related diseases. Although the current study lays a foundation for such future studies, it bears the limitations of not having extensive molecular and histological comparisons between the human and mouse NLD.

Nearly 20% of newborn infants have CNLDO, often with epiphora, dacryocystitis, and conjunctivitis. In the majority of CNLDO cases, the obstruction of the NLD is caused by a delay or failure of regression of the Hasner membrane, which mostly opens within 1 year of birth. Currently, it is not known what proportion of CNLDO is attributed to genetic causes. Although *Prickle 1* mutants manifest some phenotypes of the CNLDO, the likelihood of *Prickle 1* mutations accounting for the disease is predictably low. Nonetheless, the conserved expression of *Prickle 1* in the mouse and human lacrimal ducts suggests the possibility of finding *PRICKLE 1* mutations in human CNLDO. Although *Prickle 1/ PRICKLE 1* is indispensable for many tissues, the observed dosage effect of *Prickle 1* hypomorphic alleles in the mouse, together with the potential compensations of multiple Prickle/PRICKLE family members, may allow conditions in which human individuals with certain *PRICKLE 1* mutations are able to survive normally, yet with CNLDO-related ocular diseases.

## Supplementary Material

Supplement 1

Supplement 2

Supplement 3

Supplement 4

Supplement 5

Supplement 6

Supplement 7

Supplement 8

## Supplementary Material

**Supplementary Movie S1**. 3D reconstruction of human primordial tear duct at GW8.
