# Supplementary material for: Malformation of Tear Ducts Underlies the Epiphora and Precocious Eyelid Opening in Prickle 1 Mutant Mice: Genetic Implications for Tear Duct Genesis
Source: Invest Ophthalmol Vis Sci. 2020 Nov 3;61(13):6. doi: 10.1167/iovs.61.13.6 (PMC7645213; doi:10.1167/iovs.61.13.6)
Supplement: Supplement 1 [file iovs-61-13-6_s001.pdf]

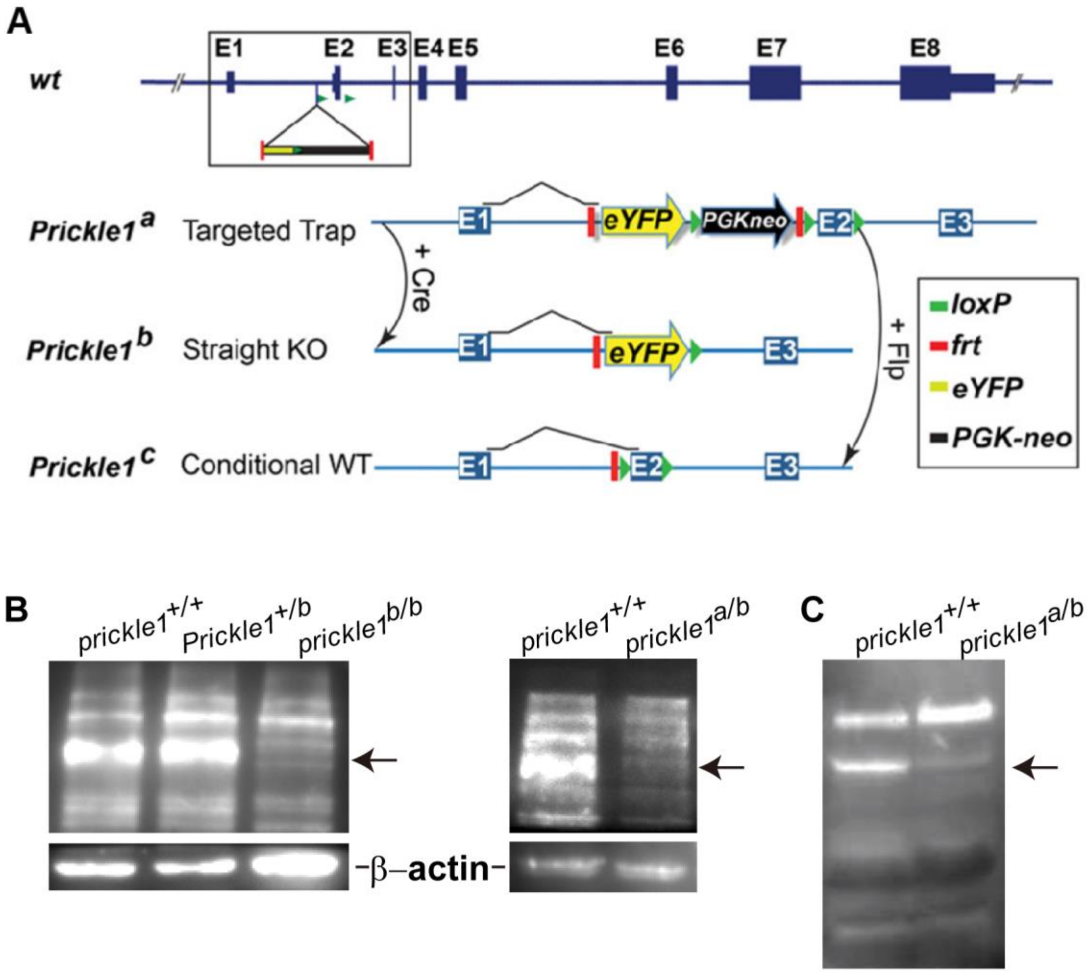

Supplemental Figure 1

Supplemental Figure 1. (**A**) Genomic structure and mutant alleles of *Prickle 1* gene (from Liu et al, 2014). The *Prickle 1*^{a} (hypomorphic) and *Prickle 1*$^{\mathbf{b}}$ (null) alleles were bred together to produce the *Prickle 1*$^{a/\mathbf{b}}$ compound mutant mice, designated as the severe *Prickle 1* hypomorphic mutants in this study. (**B**) Immunoblot of tissue extracts from E13.5 embryonic hindpaws was probed with a customized polyclonal Prickle 1 antibody. Prickle 1 was not detectable in the *Prickle 1*$^{\mathbf{b/b}}$ and only weakly expressed in the *Prickle 1*$^{a/\mathbf{b}}$ mutants (from Liu et al., 2014). (**C**) Weak Prickle 1 expression in P1 brain cortex of the severe *Prickle 1* hypomorphic mutants. Arrows points to the expected size of the Prickle 1 protein. Based on immunoblot, Prickle 1 expression was estimated as less than 25% of the wild type levels.
