# Supplementary material for: Malformation of Tear Ducts Underlies the Epiphora and Precocious Eyelid Opening in Prickle 1 Mutant Mice: Genetic Implications for Tear Duct Genesis
Source: Invest Ophthalmol Vis Sci. 2020 Nov 3;61(13):6. doi: 10.1167/iovs.61.13.6 (PMC7645213; doi:10.1167/iovs.61.13.6)
Supplement: Supplement 2 [file iovs-61-13-6_s002.pdf]

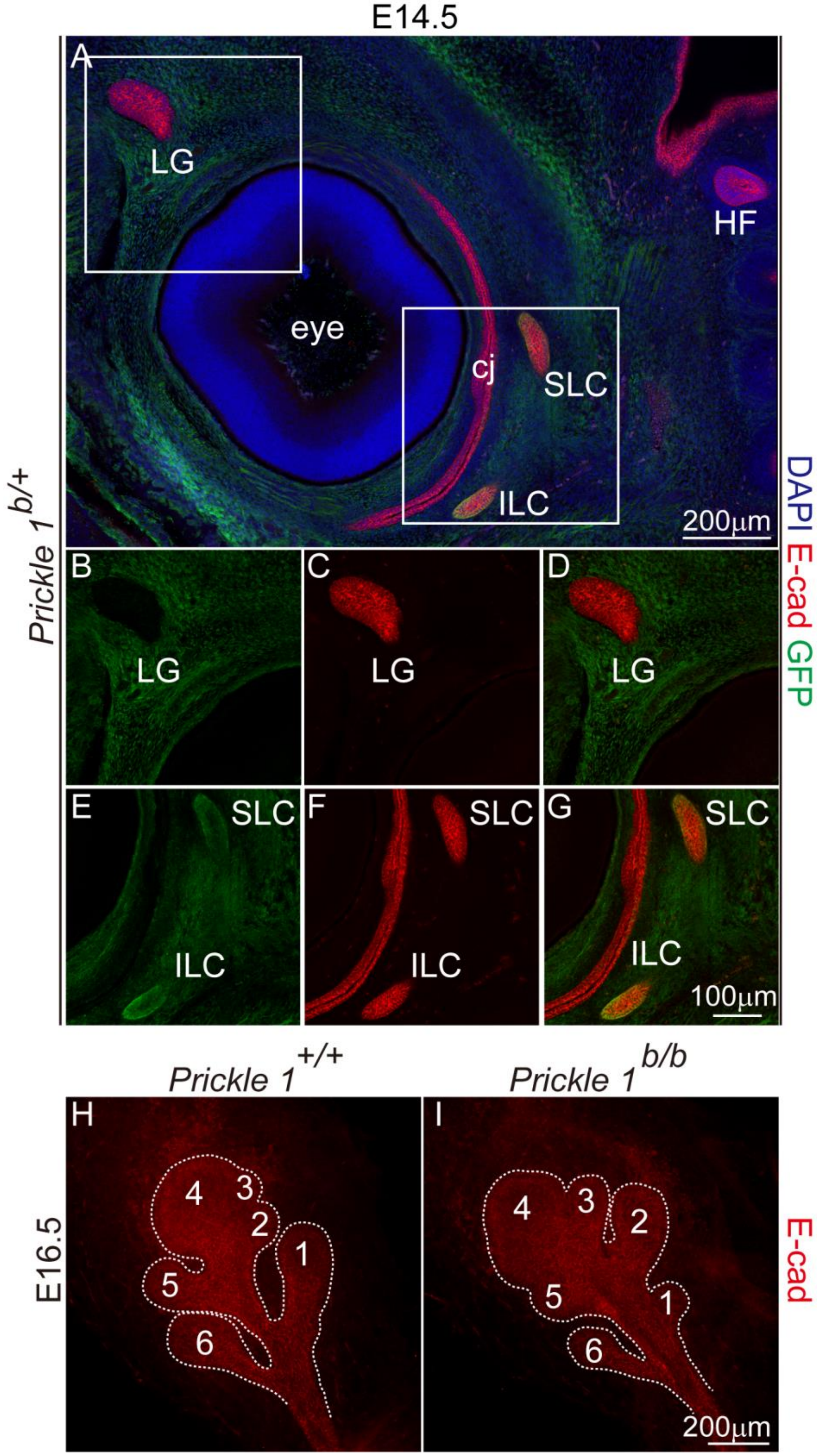
E14.5
A
LG
HF
eye
cj
SLC
ILC
200μm
Prickle 1 b/+
DAPI E-cad GFP
B
LG
C
LG
D
LG
E
SLC
ILC
F
SLC
ILC
G
SLC
ILC
100μm
Prickle 1 +/+
Prickle 1 b/b
H
4
3
2
1
5
6
I
4
3
2
5
1
6
E16.5
E-cad
200μm

Supplemental Figure 2. (A-G) Parasagittal section of a *Prickle 1*$^{b/+}$ heterozygous eye ball at E 14.5. Prickle 1 is expressed in the lacrimal canaliculi but not in the lacrimal gland . Green, Prickle 1 reporter GFP stained with anti-GFP antibody. Red, E-cadherin staining. LG, lacrimal gland; cj, conjunctiva; SLC, superior lacrimal canaliculus; ILC, inferior lacrimal canaliculus. Boxed area in the upper left corner of **(A)** is magnified in **(B-D)** in separate channels. Boxed area in the lower right corner of **(A)** is magnified in **(E-G)** in separate channels. **(H)**, A wild-type E16.5 lacrimal gland stained with E cadherin. **(I)**, A mutant E16.5 lacrimal gland stained with E cadherin. Branches of lacrimal gland are labeled with numbers.
