# Supplementary material for: Malformation of Tear Ducts Underlies the Epiphora and Precocious Eyelid Opening in Prickle 1 Mutant Mice: Genetic Implications for Tear Duct Genesis
Source: Invest Ophthalmol Vis Sci. 2020 Nov 3;61(13):6. doi: 10.1167/iovs.61.13.6 (PMC7645213; doi:10.1167/iovs.61.13.6)
Supplement: Supplement 3 [file iovs-61-13-6_s003.pdf]

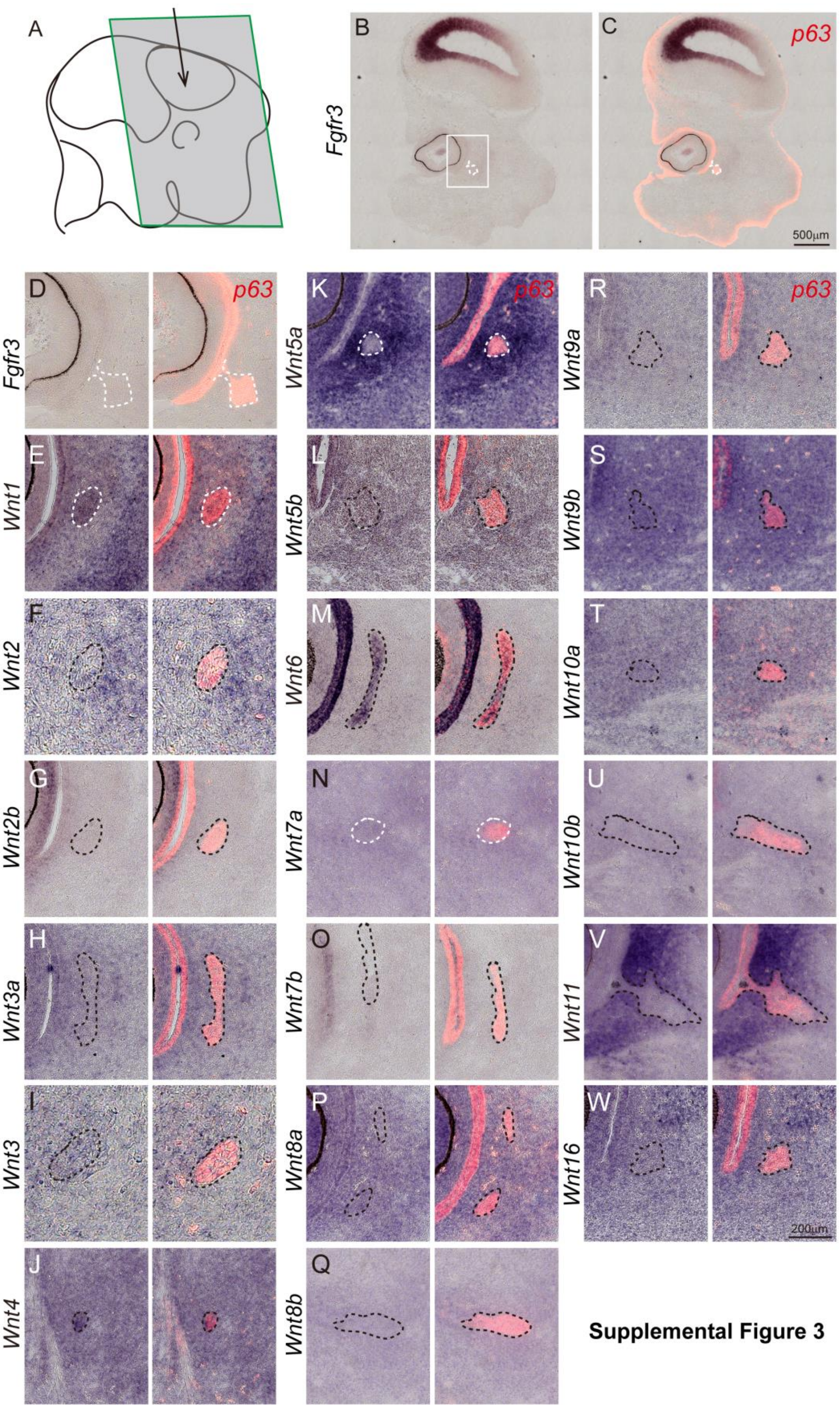

Supplemental Figure 3

Supplemental Figure 3. Expression of *Fgfr3* and Wnt family members. Embryos are at E11. Dashed lines indicate tear duct. P63 immunostaing (pink) was performed after in situ hybridization (ISH). (A) Sectioning plane. (B) *Fgfr3* was expressed in lateral ventricle, but not in tear duct. (C) p63 immunostaining on *Fgfr3* ISH. Boxed areas are magnified in D. (E-W) Expression of all 19 *Wnt* family members. Only micrographs of tear duct region are shown.
