# Supplementary material for: Malformation of Tear Ducts Underlies the Epiphora and Precocious Eyelid Opening in Prickle 1 Mutant Mice: Genetic Implications for Tear Duct Genesis
Source: Invest Ophthalmol Vis Sci. 2020 Nov 3;61(13):6. doi: 10.1167/iovs.61.13.6 (PMC7645213; doi:10.1167/iovs.61.13.6)
Supplement: Supplement 4 [file iovs-61-13-6_s004.pdf]

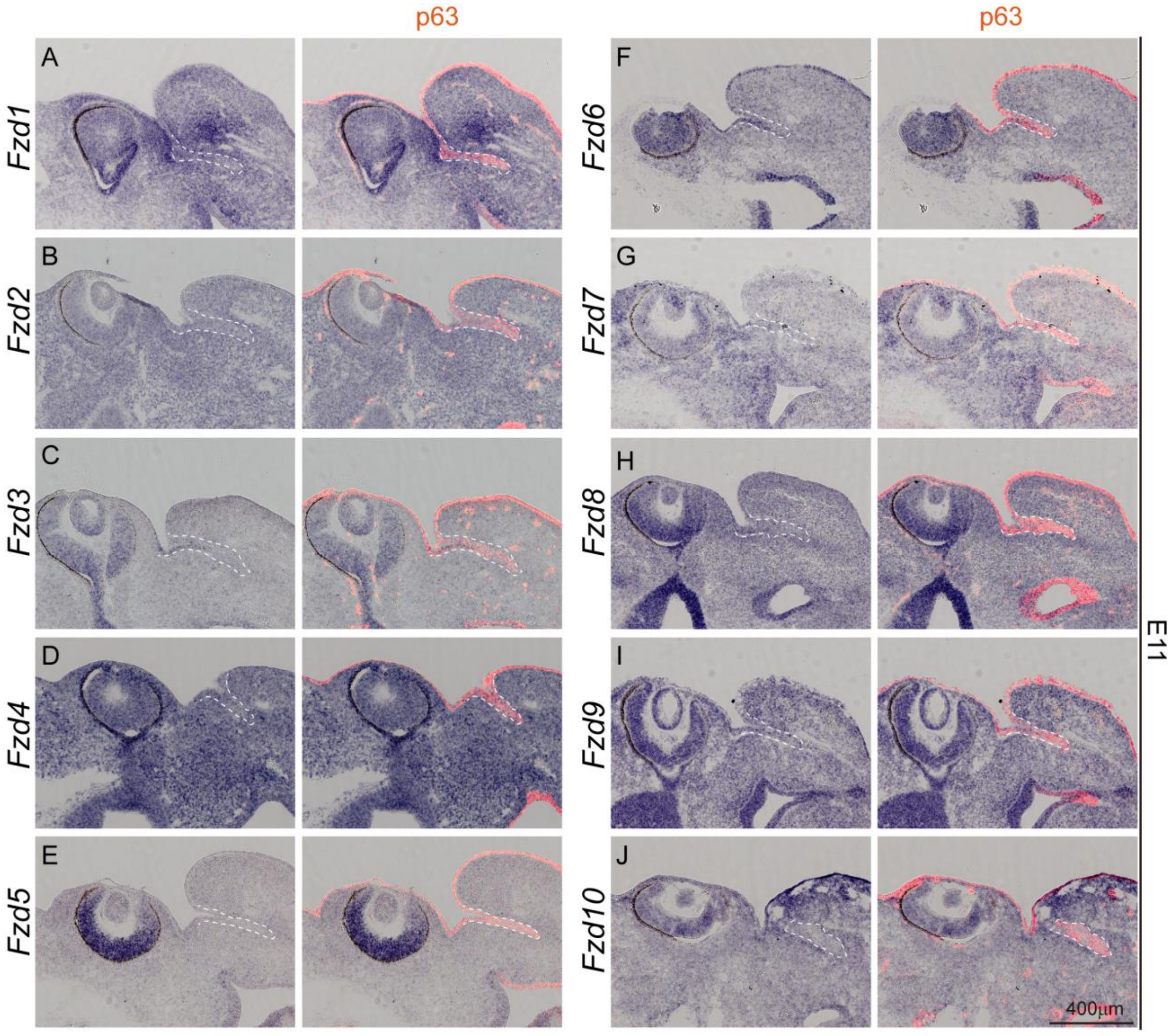

Supplemental Figure 4

Supplemental Figure 4. Expression of *Frizzled* family members. (A-J) Horizontal sections. Schematic section plane is illustrated in Figure 5S. Dashed lines indicate tear duct in each panel. Same experiments were performed on all panels as described in Supplemental Figure 2.
