# Supplementary material for: Malformation of Tear Ducts Underlies the Epiphora and Precocious Eyelid Opening in Prickle 1 Mutant Mice: Genetic Implications for Tear Duct Genesis
Source: Invest Ophthalmol Vis Sci. 2020 Nov 3;61(13):6. doi: 10.1167/iovs.61.13.6 (PMC7645213; doi:10.1167/iovs.61.13.6)
Supplement: Supplement 5 [file iovs-61-13-6_s005.pdf]

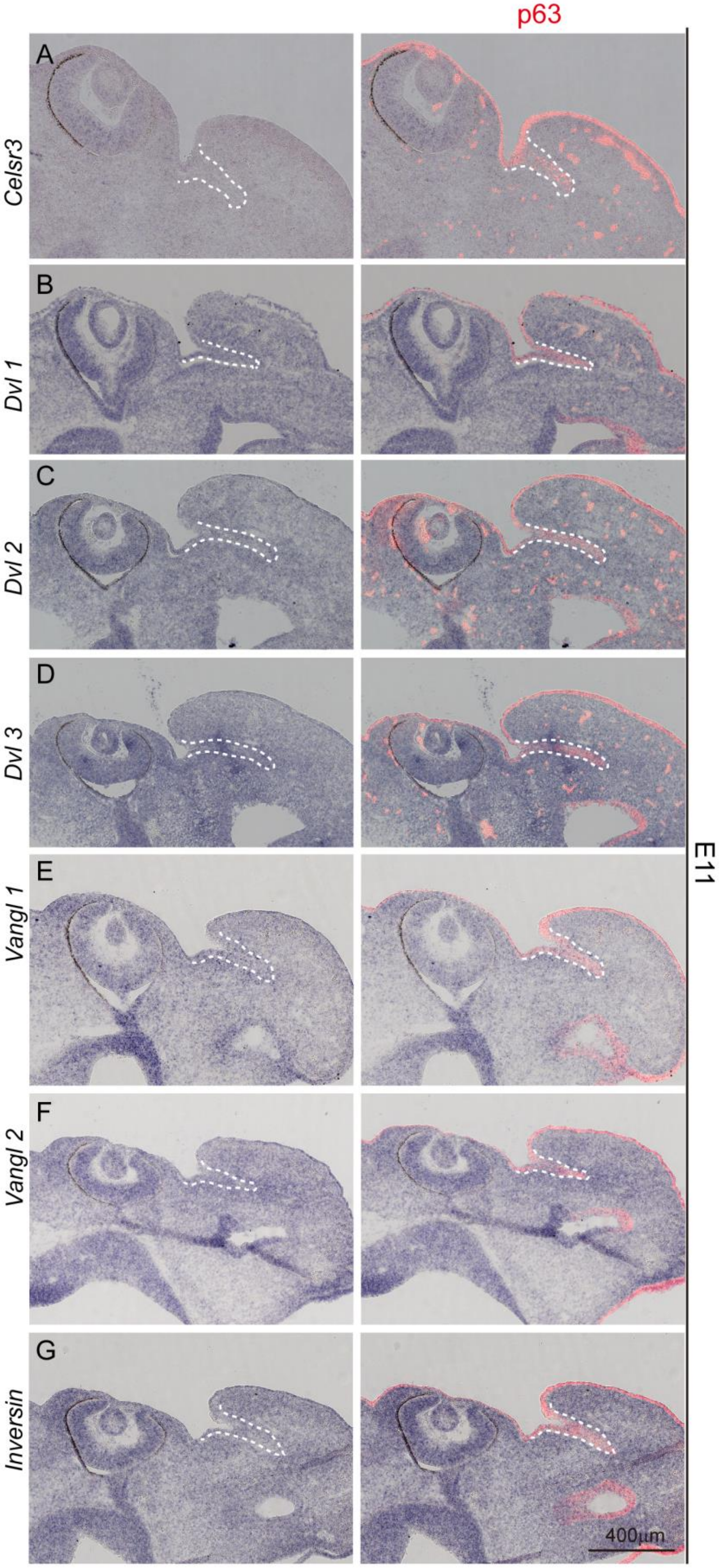

Supplemental Figure 5

Supplemental Figure 5. Expression of *Celsr*, *Dvl* and *Vangl* family members and *inversin.* Same experiments were performed on all panels as described in Supplemental Figure 2. (A) *Celsr 3*/p63. (B-D) *Dvl* (1-3) /p63. (E, F) *Vangl* (1, 2) /p63. (G) *Inversin*/p63.
