# Supplementary material for: Malformation of Tear Ducts Underlies the Epiphora and Precocious Eyelid Opening in Prickle 1 Mutant Mice: Genetic Implications for Tear Duct Genesis
Source: Invest Ophthalmol Vis Sci. 2020 Nov 3;61(13):6. doi: 10.1167/iovs.61.13.6 (PMC7645213; doi:10.1167/iovs.61.13.6)
Supplement: Supplement 6 [file iovs-61-13-6_s006.pdf]

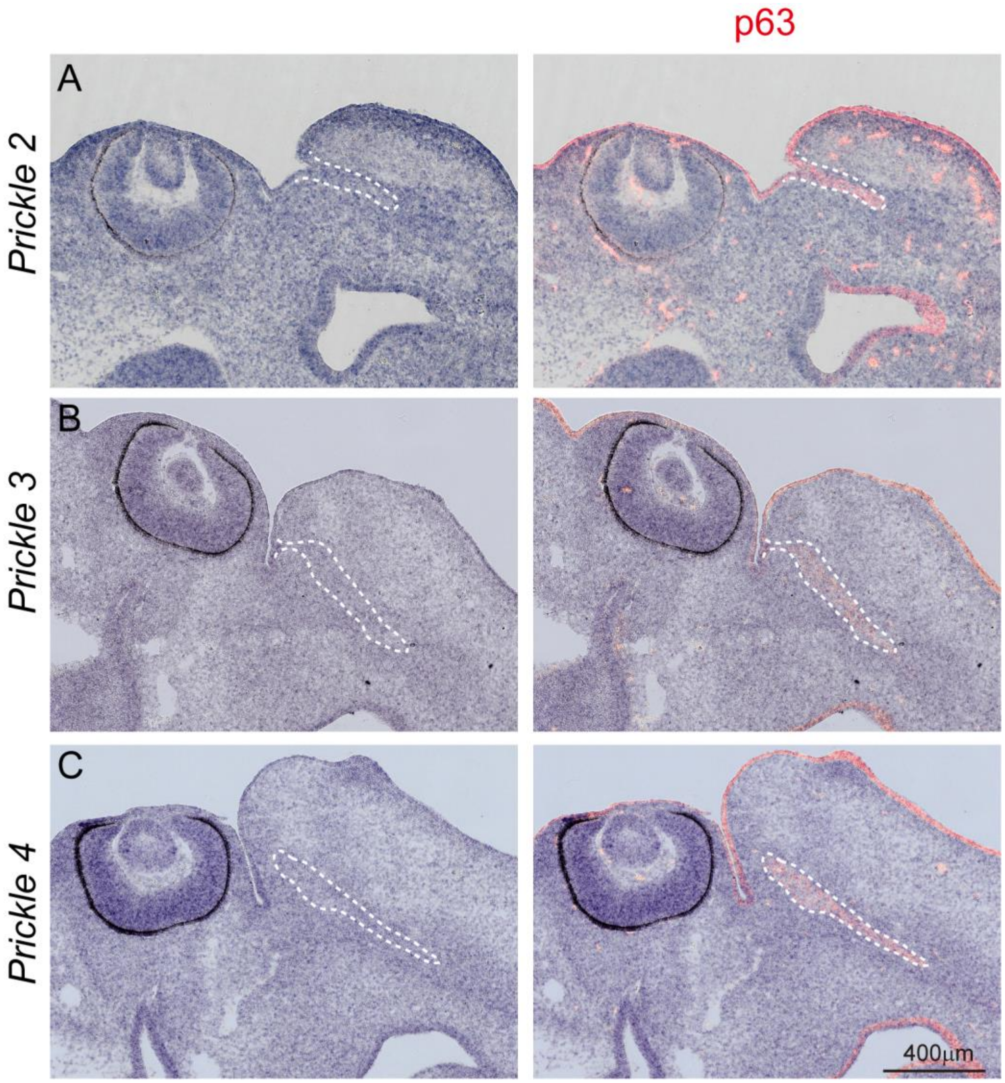

Supplemental Figure 6

Supplemental Figure 6. Expression of *Prickle* family members. Same experiments were performed on all panels as done for Supplemental Figure 2. (A) *Prickle 2*/p63. (B) *Prickle 3*/p63. (C) *Prickle 4* /p63.
